# Letter Distortion Mapping in Amblyopia: Spatial Patterns, Stability, and Relationship to Visual Acuity

**DOI:** 10.1167/iovs.66.15.65

**Published:** 2025-12-22

**Authors:** Haneieh Molaei, Reza Abbas Farishta, Reza Farivar

**Affiliations:** 1 McGill Vision Research Unit, Department of Ophthalmology & Visual Sciences, Montréal General Hospital, Montreal, Quebec, Canada; 2 Research Institute of McGill University Health Centre, Montreal, Quebec, Canada

**Keywords:** amblyopia, letter distortion, spatial mapping, visual acuity, perceptual distortion

## Abstract

**Purpose:**

To investigate whether letter-based perceptual distortions in amblyopia follow spatially consistent patterns across different letters and to determine if these spatial distortion maps are letter specific or reflect a common underlying spatial organization of visual distortion in the amblyopic eye.

**Methods:**

Twenty-one individuals with amblyopia completed a distortion mapping task using the letters A, D, and E, shown at 36 visual field locations. Each letter was first viewed with the fellow eye and then with the amblyopic eye. Participants reported distortions, which were recorded to generate binary spatial maps. The task was repeated over three sessions to assess within-subject consistency, and spatial correlations were analyzed across letters and subjects.

**Results:**

Letter distortions were reported by 95% of participants and remained consistent across sessions. Within subjects, spatial distortion maps were significantly correlated across letters in 62% of cases (*P* ≤ 0.028), suggesting shared spatial patterns. However, across subjects, maps were largely uncorrelated, indicating individualized distortion profiles. No single letter consistently showed more distortion across the group, χ^2^(2) = 1.279, *P* = 0.5. A strong positive correlation was found between interocular visual acuity difference and overall distortion intensity (*r* = 0.70, *P* < 0.001), consistent across all letters.

**Conclusions:**

Letter distortions in amblyopia are highly prevalent, reliable, and spatially organized within individuals but idiosyncratic across subjects. These distortions correlate strongly with visual acuity loss, highlighting their potential as a clinically valuable and perceptually relevant measure for characterizing amblyopic visual dysfunction.

Amblyopia is a neurodevelopmental disorder of the visual system, characterized by reduced visual acuity (VA) in one or both eyes, caused by abnormal visual experience during early childhood. Among the deficits associated with amblyopia, such as low VA, reduced contrast sensitivity, impaired stereoacuity, and binocular dysfunction, *perceptual distortion* is particularly critical to study because (1) it cannot be understood or identified through VA measurement, even after VA improvement,^1^^–^^7^ and (2) it directly affects both binocular and three-dimensional vision in amblyopia.^6^^,^^9^^–^^12^ Visual distortions are multifactorial, varying in space, spatial frequency, and orientation, as we^13^ and others^14^^–^^17^ have shown. Therefore, evaluation and incorporation of spatial distortions can be valuable for understanding and characterizing amblyopia clinically.

The earliest evidence of perceptual distortion in amblyopia came from studies using letter charts.^18^^,^^19^ However, these studies examined only the abnormal perception of letters in the amblyopic eye (AE) and did not explore the intensity or types of distortions in detail. Letter grids have rarely been used as stimuli to assess perceptual distortion across the visual field (VF) in amblyopia^16^^,^^20^^–^^22^; instead, they have primarily been employed to measure the VA of the AE using Snellen, Early Treatment Diabetic Retinopathy Study (ETDRS), or other types of charts.^23^^–^^30^ Other simple stimuli instead became the focus for extracting perceptual distortion, including (1) displacement, assessed using blobs or dots as stimuli,^7^^,^^8^^,^^31^^–^^33^ and (2) orientation or spatial frequency (SF) distortion, captured using Gabor patches or gratings at various spatial frequencies.^14^^,^^34^^–^^39^ However, letters offer a practical and clinically familiar tool for identifying perceptual distortions, as they are easy to recognize and report—similar to their use in Snellen and ETDRS charts—although they have rarely been used to systematically assess the nature or pattern of distortion in amblyopia. Therefore, a gap in our understanding of the nature of letter distortion in the AE remains to be addressed.

In understanding perceptual distortions of letters, it could be argued that the legibility—whether a letter is easy or hard to read, which has been extensively investigated—could be considered a form of visual distortion. However, visual distortion of letters is not entirely related to legibility in the perception of distorted letters for several reasons. First, the size of a letter has a clear role in the readability of letters. Some claim that smaller, easy-to-read letters (e.g., A, U, or P) are more legible than larger letters that are difficult to read (e.g., B, F, or S),^26^^,^^39^^,^^40^ whereas others emphasize the importance of letter size for accurate perception.^16^^,^^20^^,^^22^^,^^27^ Compared to this mixed evidence on readability, the influence of size on visual distortion appears more consistent—visual distortion of letters undoubtedly depends on the size of the stimulus.^16^^,^^21^^,^^41^

Second, Mathew et al.^42^ found that the difficulty of letter legibility is the same for both the AE and the fellow eye (FE), indicating that low readability of a letter is not necessarily attributable solely to amblyopia-related deficits. In other words, legibility may help reveal distorted perception in amblyopia but perception of distortion in letters cannot be explained by just legibility.

Third, even if we set aside the study by Mathew and his team,^42^ one can still argue that an easy-to-read letter may exhibit significant distortions, yet its overall form remains recognizable; for example, a shaky, tremulous, or fuzzy A could still be read as an A, and the patient may not have any means of reporting the distortion they perceive. On the other hand, a difficult-to-read letter might have minor alterations that drastically change its perception, possibly causing it to be mistaken for a different letter—for example, an extra faded horizontal or vertical line turning an F into a P or an E. This suggests that readability alone cannot reveal the extent of distortion in a letter or deeper perceptual irregularities. Thus, reading errors of letters alone may not inform us whether a letter is perceived as distorted or not.

Fourth, the correlation between VA loss and legibility is well established,^21^^,^^22^^,^^24^^,^^26^^,^^42^^,^^43^ as VA measurement is based on the ability to read letters, but, in contradistinction, there is no meaningful correlation between VA loss and visual distortion in amblyopia in mainstream findings.^1^^–^^7^ Our recent multifactorial mapping of distortions also corroborates this absence of a relationship between the presence of distortions and VA loss.^13^

There may be some overlap between the concepts of legibility and visual distortion, as the root cause of each remains unknown. Alexander et al.^44^ found that circular or curvy letters are more difficult to identify than angular letters. For example, participants sometimes confuse the letter D with O or C, or the letter B with S or G, among others.^26^^–^^28^^,^^42^^,^^45^^,^^46^ This suggests that letter misperception can be attributed either to the low legibility of the letter—such as the letter B being among the more difficult to read letters—or to certain types of deformation in the letter that affect overall letter perception.

Another overlapping factor between legibility and distortion is the position of letters across the VF. For legibility, the location of a letter in a word or retinal location influences how easily it is read.^42^^,^^47^ For example, letters at the end of a line are more likely to be misread than those at the beginning, even when using Sloan letters in the ETDRS chart, which are designed to have nearly identical legibility.^48^ Likewise, even charts with the same set of letters presented in different sequences cannot be assumed to be equivalent in terms of identifiability.^40^ Importantly, crowding effects—often viewed as a form of perceptual distortion—also depend on letter position, being stronger in some parts of the line or field than others.^42^

From a neurophysiological perspective, positional uncertainty and receptive field size both increase toward the visual periphery.^49^^,^^50^ Larger and less spatially precise receptive fields reduce localization accuracy and spatial resolution, leading to greater positional uncertainty and increased crowding in peripheral vision. These physiological constraints likely contribute to differences in letter legibility between foveal and peripheral vision, even in healthy observers.^28^ Therefore, it would hardly be surprising if this positional dependence also occurred in individuals with amblyopia. These observations suggest that, although legibility and distortion remain distinct concepts, they share common influencing factors that can affect both phenomena.

We have recently assessed the role of location—the retinal/VF position of the stimuli—in visual distortion in amblyopia,^13^ and other studies have also explored how spatial position influences distortion.^17^^,^^47^^,^^51^^–^^53^ This can offer new insights into distorted letter perception in amblyopia. For example, testing an easy-to-read letter at different locations within the VF can help reconstruct a perceptual distortion map for the AE. These individual maps can then be compared to determine whether the distortion pattern depends on the letter type (content) or whether it is a space-based map independent of content.

In this study, we assessed letter distortion across the VF and developed a quick, easy-to-use method for measuring perceptual distortions. To isolate and evaluate distortion, we used easy-to-read letters of medium size with high contrast, minimizing the influence of legibility and size on the measurements. Letters were placed at different locations within the VF, with the focus on identifying distortions of varying intensity—from small to large—across different spatial positions, regardless of the actual readability of the letters at each location. By holding readability constant across letters, this design also allowed us to evaluate whether distortion maps depended on letter identity (content-based) or remained consistent across letters (space-based). In other words, even if a subject could correctly identify a letter, any distortion in its visual appearance was still captured.

Other aspects of the distortion map were also assessed, including (1) whether the letter distortion map was evenly distributed across the VF, (2) which letter was dominantly distorted within a subject and across subjects, (3) whether letter distortion maps were spatially correlated within a subject and across subjects, and (4) whether lower VA was associated with more intense distortion across subjects.

## Methods

### Participants

A total of 21 individuals with amblyopia participated in the study, including seven with strabismus, nine with anisometropia, and five presenting a combination of both conditions. Participants wore their most recent prescriptions to isolate the effects of amblyopia. The study adhered to the tenets of the Declaration of Helsinki and was approved by the Research Ethics Board of the McGill University Health Centre. All participants were fully informed about the nature of the experiment and provided written consent prior to participation.

Participants were included if they demonstrated an interocular acuity difference of ≥0.2 logMAR without a history of occlusion or atropine penalization treatment, or ≥0.1 logMAR if they had undergone treatments such as patching or surgery. Individuals with severe visual acuity impairment (worse than 1.3 logMAR in either eye) were generally excluded. One participant with slightly poorer acuity was initially included because they could perform the task reliably; however, analyses were re-run excluding this participant, and the results remained consistent. The clinical characteristics of the participants are summarized in the Table.

**Table.** Clinical Parameters of the Amblyopic Participants

			VA (logMAR)^*^						
Subject	Age (Y)	Amblyopic Eye	Right Eye	Left Eye	Type	Intervention	Stereo	Deviation	Refractive Error
A1	36	R	0.1	0	A	P	Not measured	—	OD +3.50/−0.25 × 155
									OS: +3.00/0.−25 × 25
A2	24	L	0.1	0.7	A	N	50s	—	No Rx
A3	30	R	0.5	0.1	S	P	Nil	2 PD	OD: −0.25/−0.50 × 153
									OS: −2.25/−0.75 × 20
A4	42	R	0.1	0	M	S & P	140s	14 PD	No Rx
A5	22	L	0.2	0.5	S	S & P	70s	3 PD	No Rx
A6	34	L	0.1	0.2	A	P	70s	—	No Rx
A7	20	R	0.1	0	S	S & P	250s	3 PD	OD: plano/−0.75 × 5
									OS: −0.25/−0.25 × 130
A8	67	L	0.1	0.6	A	N	Nil	—	OD +0.25/−0.25 × 100
									OS: +0.50/−0.25 × 70
A9	70	R	1.3	0.2	S	S & P	Nil	—	OD: +5
									OD: +4.75
									Add: +2.50
A10	57	L	0	1	A	N	Nil	—	OD: −1.00/−1.00 × 73
									OS: −1.00/−1.25 × 65
A11	38	R	CF	0	S	P	Nil	10 PD	No Rx
A12	47	L	−0.1	1.2	M	N	Nil	Not reliable	OD –4.00
									OS: −19.00
A13	24	L	0	0.2	A	P	Nil	—	OD: −4.00
									OS: +2.00/−1.25 × 75
A14	28	L	0	0.4	M	P	250 s	10 PD	OD: −1.75/−0.25 × 160
									OS: +2.00/−2.00 × 180
A15	28	R	0.8	−0.16	A	P	Nil	—	No Rx
A16	30	L	0.1	0.3	S	S & P	Nil	6 PD	OD: +3.75/−0.50 × 170
									OS: +5.00
A17	32	L	0.1	0.6	M	P	800 s	10 PD	No Rx
A18	60	L	0.1	0.4	A	N	Nil	—	OD: planot/−0.75 × 70
									OS: +3/−1.75 × 70
									Add: +2.25
A19	37	R	0.2	0.1	A	N	250 s	—	OD: −1.00/−0.50 × 180
									OS+2.75/2.75 × 175
A20	24	R	0.2	0	A	N	—	—	OD: +1.00/−1.00 × 10
									OS: +0.50
A21	35	R	0.1	0	S	S & P	Nil	5 PD	No Rx

The VA columns display the best-corrected VA of each eye; the CF entry in the right-eye column for participant A11 indicates “counting fingers at a distance of 40 cm.” The Type column indicates the type of amblyopia: A, anisometropia; S, strabismus; M, both anisometropia and strabismus mixed. In the Intervention column, P indicates patching; N, no intervention; and S, surgery. R, right eye; L, left eye; OD, right eye; OS, left eye; Rx, prescription; PD, prism diopters.

* Acuity tested with glasses.

### Experimental Design and Stimuli

**Figure 1.**
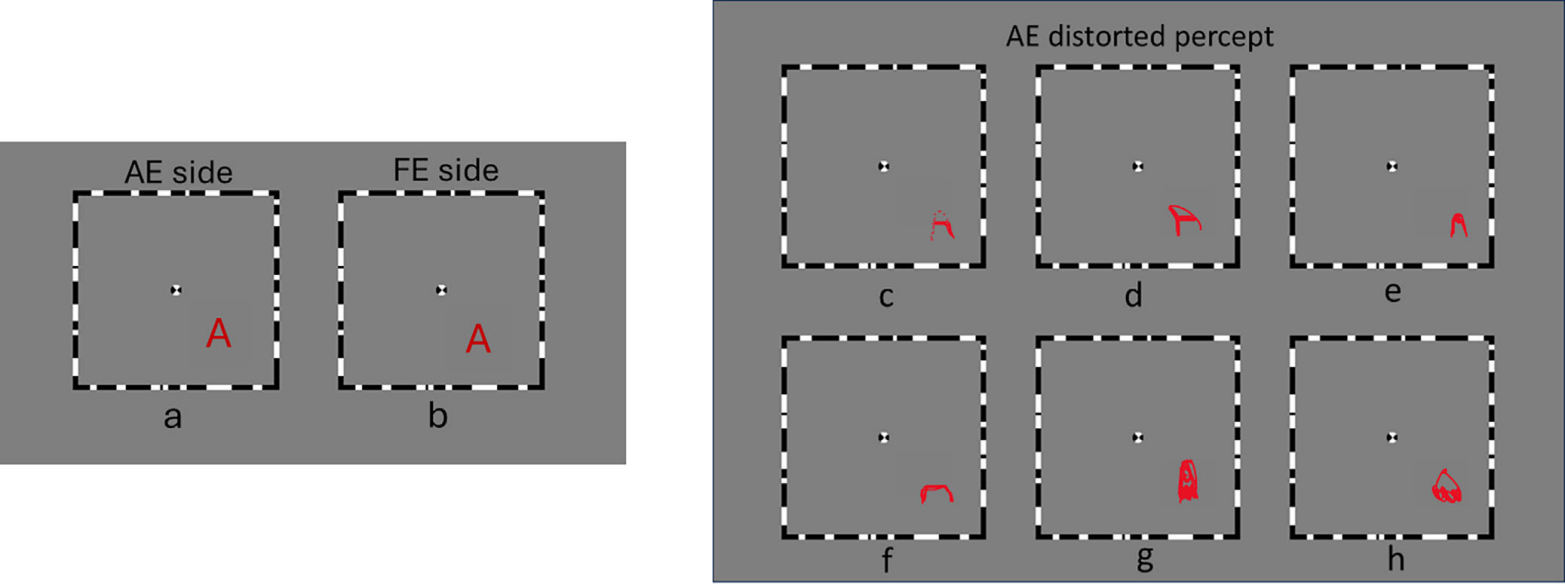
**Stimuli presentation and perceptions in AE.** (**a**, **b**) Stimuli presented to the AE and FE on separate sides of the screen. (**c**–**e**) Actual distorted perceptions as drawn by amblyopic participants, where the overall form of the letter A was preserved: fragmented and partially incomplete A (**c**), an A with exaggerated curvature (**d**), and an A with a smaller, nearly filled hole and that is slightly curvy on the top (**e**). (**f**–**h**) Distortions where the form of the letter A was not preserved, making it unrecognizable to the subjects.

The visual stimuli were presented on a gamma-corrected ASUS liquid-crystal display (LCD) monitor (ASUS, Taipei, Taiwan) to ensure linearity of the luminance profile. The screen brightness was set to 30.75 cd/m^2^, with a resolution of 1280 × 1024 pixels and a refresh rate of 75 Hz. The experiment was programmed in Python 3.8 using PsychoPy 2021.2.3 and related standard dependencies. The stimulus display featured two borders of alternating black-and-white lines with a fixation point at the center. These stimuli were shown in two separate windows on either side of the monitor, as illustrated in Figures 1a and 1b, to enable a direct comparison of perception between the AE and the FE. The methods used here were almost identical to those described in our recent study.^13^

**Figure 2.**
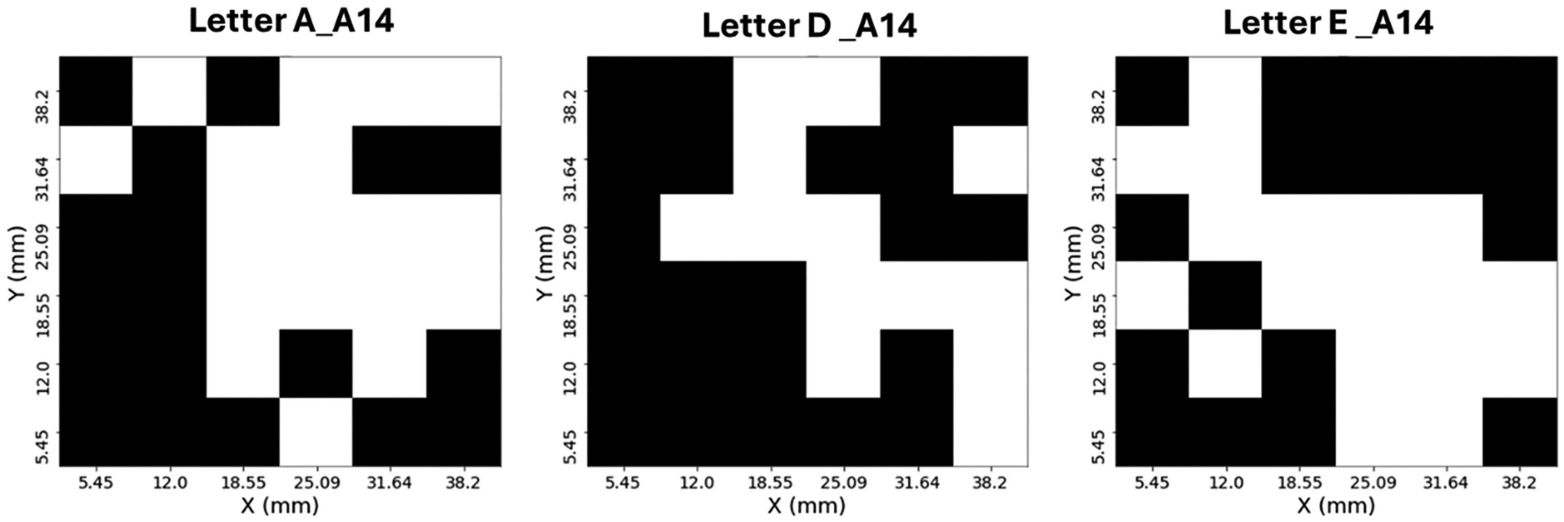
**Example of binary letter-distortion maps in an amblyopic participant distortion map for three letters in subject A14.** Each grid represents the 6 × 6 array of VF locations (5° × 5° region) tested in the AE of one subject (A14). *Black squares* indicate locations where a distortion was reported (value = 1), and *white squares* indicate veridical perception (value = 0). Panels show the distortion maps obtained for the letters A, D, and E. These binary maps visualize the spatial pattern of perceived distortions across the VF, where clusters of *black cells* reflect regions of locally distorted perception.

Each window covered approximately 5° of the VF when subjects were seated 50 cm from the screen. The internal 6 × 6 grid itself spanned 4.5°, with each cell subtending 0.75° of visual angle. An additional 0.25° border on each side (left and right) was included to create the alternating black-and-white frame, resulting in a total coverage of 5° of visual angle. A letter (e.g., A, D, or E) was randomly positioned in one of these 36 cells, and the same presentation pattern was repeated in the other window (see Figs. 1a, 1b). Subjects fixated on the central point—covering 0.25° of the VF—in the FE window to observe the type, form, shape, size, and boldness of the letter. They then covered their FE using a patch, fixated on the center of the AE window, and perceived the letter with their AE to compare it to their FE perception. Any detected difference in size, shape, boldness, or other features was classified as a distorted perception. To illustrate their perception, subjects were asked to draw the distorted letter by hand on paper while viewing with their fellow eye, as shown in Figures 1c to 1h. Drawings in Figures 1c to 1e represent distortions where the overall form of the letter A was preserved, meaning subjects could still recognize it as the letter A despite the distortion. In contrast, drawings in Figures 1f to 1h depict distortions where the form of the letter A was no longer preserved, and subjects were unable to identify it as A. The procedure was repeated 35 times for the remaining 35 cells in the 6 × 6 grid, resulting in a total of 36 trials for the letter A. In each trial, the output was recorded as “yes” for a distortion or “no” for an identical perception to the FE, resulting in a binary map of 36 values, with “1” indicating distortion and “0” indicating no distortion. This map is referred to as the binary distortion map for the letter A. The same procedure was then repeated for the letters D and E, providing each participant with three binary distortion maps, one for each letter (A, D, or E) (see Fig. 2). All three letters are recognized as easy-to-read letters.^27^^,^^42^^,^^45^^,^^46^

The entire process described above was completed in a single session, which served as a training session. This session facilitated interaction between the participant and the data collector, clarifying the task requirements. Data collected during this training session were used only for practice and were not included in the final analysis. The data collector remained with the participants throughout all sessions to ensure correct task performance and provide support as needed. Additionally, the data collector documented verbal descriptions provided by participants about perceived differences between the AE and FE, alongside the key (space bar) on the keyboard used to advance to the next trial.

The data collector remained with the participants throughout all sessions to ensure correct task performance and provide support as needed. Additionally, the data collector documented verbal descriptions provided by participants about perceived differences between the AE and FE, alongside keypresses on the space bar used to advance to the next trial. Each session lasted approximately 30 minutes. To assess the reliability and repeatability of the distortion map and experimental design, the entire task was repeated on three separate days for each participant, totaling 1.5 hours. Participants were permitted to rest as needed throughout the sessions.

### Quality Control

To assess stability of measurements and ensure the reliability of the distortion maps, a test-retest reliability analysis was conducted across three separate sessions. The intraclass correlation coefficient (ICC)^54^ was used to quantify the level of agreement between repeated measurements. According to established thresholds, ICC values below 0.5 indicate poor reliability, values between 0.5 and 0.75 indicate moderate reliability, values between 0.75 and 0.9 indicate good reliability, and values above 0.9 indicate excellent reliability.^55^ Fixation stability was verified using an EyeLink 1000 Plus (SR Research, Ottawa, ON, Canada), and deemed as stable in participants during a 25-second test period where we found the fixation was comparable to controls. This comparison was based on a separate fixation test conducted between amblyopic participants and a group of control observers, used only to establish a baseline for normal fixation stability. The control data were collected independently from the main experiment and were not included in the distortion analyses. This finding confirms that amblyopic participants maintained stable fixation comparable to controls, consistent with previous studies reporting short-duration fixation stability in amblyopia.^33^^,^^56^^,^^57^

### Statistical Analysis

All statistical analyses were performed using Python 3.8 and JASP 0.18. Within-subject consistency of distortion maps across sessions was assessed using the ICC to evaluate test–retest reliability. The spatial correspondence between binary distortion maps was quantified using Spearman's rank correlation coefficient (ρ), computed for each pair of letters (A–D, A–E, and D–E) within subjects. Significance was determined using two-tailed tests with α = 0.05.

To examine potential commonalities in distortion patterns across subjects, pairwise correlations were performed between distortion maps from all subject pairs for each letter type. A Bonferroni correction was applied to control for multiple comparisons (corrected α = 0.05/210 per letter). To determine whether certain letters were more distorted than others within subjects, the coefficient of variation (CV) and ratio analysis were used to quantify distortion dominance. Group-level differences in distortion intensity across letters (A, D, and E) were analyzed using a nonparametric Friedman test. The relationship between interocular VA difference (logMAR) and the extent of distortion was evaluated using Spearman's or Pearson's correlation coefficients, depending on the normality of data assessed by the Shapiro–Wilk test. Statistical significance was set at *P* < 0.05, and all tests were two-tailed.

## Results

### Reliability of Distortion Measurements

Test–retest analyses confirmed that the extracted distortions from letters across the VF were consistent across 21 subjects tested on three different days. Almost all participants (20 out of 21) reported some form of distortion in their perception (e.g., smaller, larger, bolder, faded, misidentified, or unidentifiable letters) across their VF. Accordingly, three binary distortion maps were generated for each participant—one for each letter (A, D, and E).

A sample of the binary distortion letter maps from one subject is illustrated in Figure 2. Each grid corresponds to the 6 × 6 test locations covering a 5° region of the VF in the AE. Black squares mark locations where the participant reported a perceived distortion, and white squares mark undistorted perceptions. Together, these maps for letters A, D, and E show that distortions tend to cluster within specific VF regions rather than appearing randomly.

We assessed letter distortions in two ways: (1) by analyzing the spatial distribution of distorted locations across the VF, which captures the pattern or structure of distortion, and (2) by measuring the distortion intensity of each letter—quantified as the total number of distorted spots across the VF—to capture the overall extent of distortion. Both analyses are quantitative and can be applied within individual subjects and across the group.

### Spatial Structure of Letter Distortion Maps: Within-Subject Correlations

**Figure 3.**
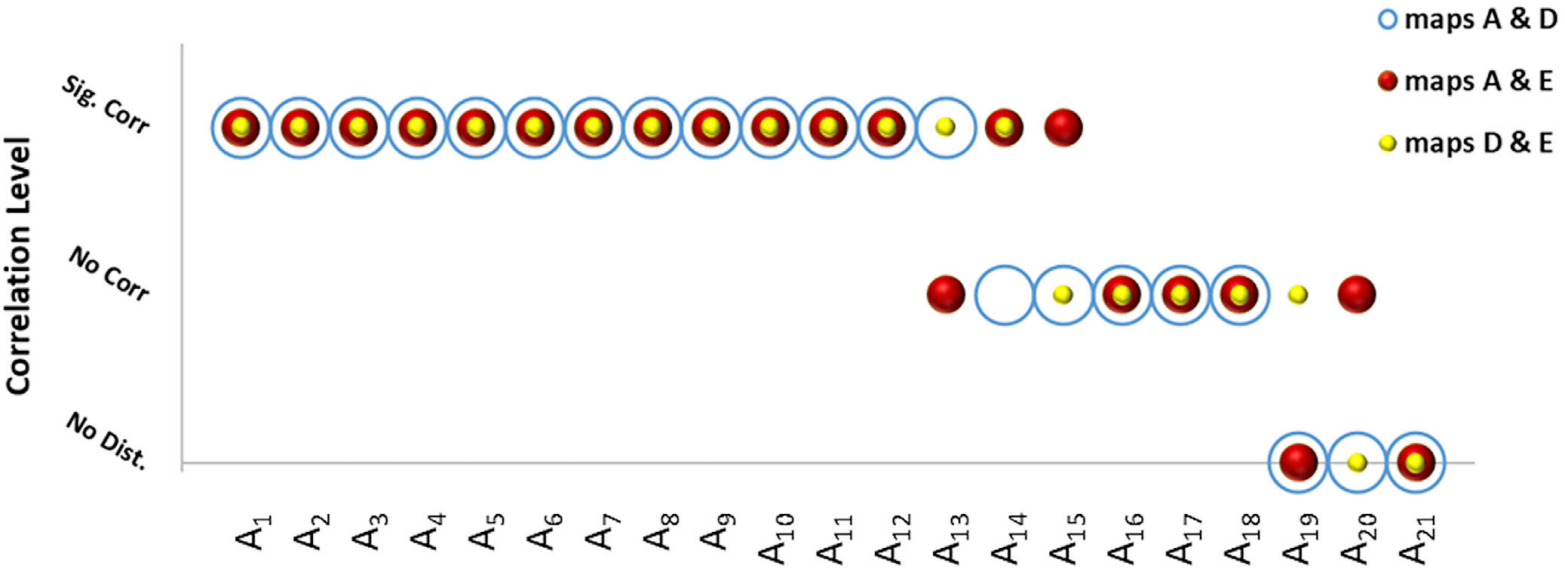
**Spatial correlation levels between letter-distortion map pairs within subjects.** Each column represents one participant (A1–A21). *Circles* indicate the three possible pairwise correlations between letter maps: A&D (*blue* *outline*), A&E (*red-filled*), and D&E (*yellow-filled*). The vertical position of each circle corresponds to the correlation outcome. Sig. Corr, significant spatial correlation; No Corr, non-significant correlation; No Dist., no distortion in one of the two maps. The figure therefore summarizes, for each participant, which letter-pair maps showed statistically reliable spatial similarity. Categorical levels are displayed for clarity but represent a continuum of correlation strengths across individuals.

Across 21 participants (63 map-pair comparisons in total)—or 20 participants (60 comparisons) when excluding A11, the participant with the poorest visual acuity—within-subject spatial correlations ranged from ρ = 0.37 to 0.8 (mean ρ = 0.52 ± 0.13, *P* values ranged from 0.001 to 0.03). Significant correlations were observed in 62% of participants for all three letter pairs (60% when excluding A11) and in 24% for one or two pairs; 14% showed no significant correlations. These proportions are reported descriptively and should not be interpreted as discrete subgroups, as correlation strength varied continuously across participants. These findings indicate that most participants exhibited systematic, qualitatively similar patterns of distortion across different letters. Figure 3 summarizes these within-subject correlations, showing for each participant which letter-pair maps were significantly or non-significantly correlated. As shown in Figure 3, no particular map pair (A–D, A–E, or D–E) dominated across participants; all three pairings had roughly equal likelihoods of exhibiting significant spatial correlations.

### Spatial Structure of Letter Distortion Maps: Across-Subject Correlations

**Figure 4.**
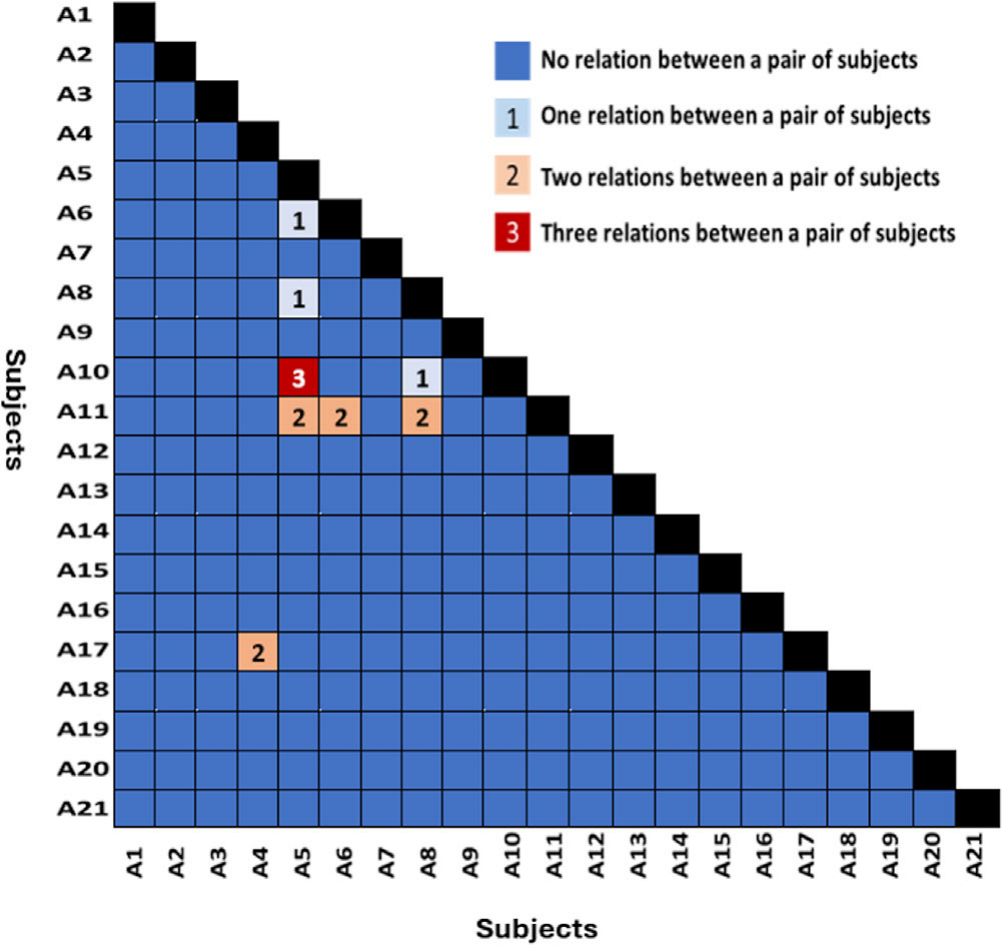
**Pairs of subjects with significant correlations across letter distortion maps.** The figure illustrates the number of significant spatial correlations in letter distortion maps (**A**, **D**, and **E**) for each pair of subjects. Each cell represents a pair of subjects, color-coded to indicate the number of correlated maps: *navy blue*, 0 maps; *light blue*, 1 map; *orange*, 2 maps; and *red*, 3 maps.

To examine whether distortion patterns were shared across participants, we analyzed all possible pairwise combinations of subject maps for each letter (Fig. 4). This resulted in 210 subject pairs per letter, or 630 total comparisons across the three letters. Only correlations that survived Bonferroni correction were considered significant. These extensive pairwise comparisons were conducted solely to confirm that distortion maps were idiosyncratic and not shared across participants, rather than to perform detailed statistical testing between individuals.

The results, summarized in Figure 4, revealed that only one pair of participants exhibited significant spatial correlation across all three letters (shown in red). Four pairs showed significant correlation in two of the three letter maps (orange; although including A11 produced these four pairs, excluding this participant did not alter the overall result), and three pairs showed correlation in one letter map (light blue). The remaining subject pairs showed no significant correlation in any of their maps (navy blue).

These findings indicate that the spatial distortion maps for the three letters (A, D, and E) are largely unique to each subject. Spatial correlations are rare across subjects, suggesting that distortion patterns are subject specific and do not generalize across individuals. However, this lack of across-participant correlation should be interpreted cautiously, as individual differences in response criterion could contribute to variability in the reported distortions. Nonetheless, the overall absence of shared spatial structure supports the view that these perceptual distortions are idiosyncratic in nature.

### Letter Distortion Dominance Within Subjects

We next examined whether certain letters tended to produce more perceptual distortions than others within individual participants. In other words, was one letter systematically more distorted across different spatial locations of the VF? For each participant, the binary distortion maps for the letters A, D, and E were converted into three scalar values representing the total distorted intensity (i.e., number of distorted locations out of 36) for each letter. Two complementary metrics were then used to assess whether one letter was dominant (i.e., distorted in more VF locations than the others):

1. Coefficient of variation—Calculated as the standard deviation divided by the mean of the three distortion counts; a CV > 50% was considered indicative of a dominant letter.
2. Ratio analysis—Calculated as the maximum distortion count divided by the total across all three letters; a ratio above 0.5 indicates that one letter accounts for more than half of the distortions.

**Figure 5.**
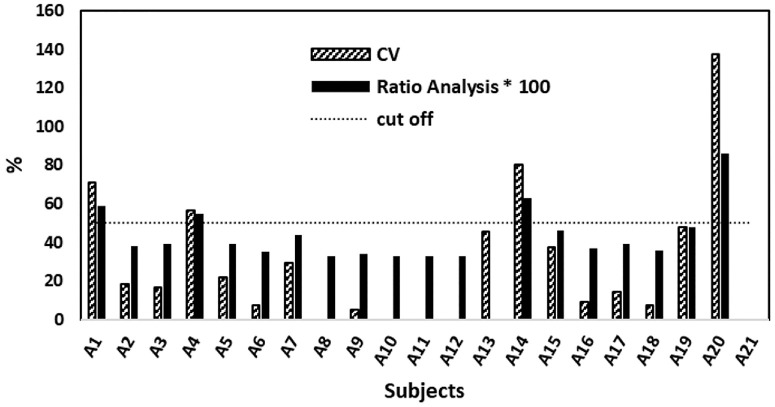
**Letter distortion dominance analysis based on CV and ratio analysis within subjects.** *Bars* show CV values (*solid blue*) and ratio values (*striped*, multiplied by 100 for visual comparison) for each subject, quantifying the imbalance in distortion across three letter maps (**A**, **D**, and **E**). The *horizontal dashed line* marks the dominance threshold (50%) used for both metrics. Subjects exceeding this threshold were considered to exhibit dominance of one letter over the others in terms of the number of distorted spots—distortion intensity—across the VF. Only four subjects (A1 and A4 with letter E, A14 with letter D, and A20 with letter A) crossed the cutoff, and, even among these, the dominant letter varied, suggesting no systematic bias toward a particular letter. For most subjects, distortion was distributed relatively evenly.

These metrics captured the uneven distribution of distortions within each participant. Figure 5 summarizes the results, displaying CV (%) and ratio analysis (×100) values for each subject. The horizontal cutoff line marks the 50% dominance threshold for both measures. Only four participants (A1 and A4 for letter E, A14 for D, and A20 for A) exceeded either threshold, indicating clear within-subject dominance (this outcome remained unchanged when A11 was excluded). Even among these few cases, the dominant letter differed across individuals. For the majority of participants, distortions were distributed relatively evenly across the three letters, suggesting that no single letter consistently drives perceptual distortion within subjects.

### Letter Distortion Dominance Across Subjects

After examining distortion dominance within individual participants, we next evaluated whether any of the three letters (A, D, or E) was consistently more distorted than the others across the group. This analysis aimed to determine whether specific letter forms systematically elicited stronger distortions at the population level, which would suggest a content-based effect or shared perceptual bias across individuals. Across the entire sample, the average distortion scores for letters A, D, and E were highly similar. A Friedman test revealed no significant difference among the three letters, χ^2^(2) = 1.279, *P* = 0.5. This contrasts with the within-subject results, where a few individuals exhibited letter dominance, and indicates that letter-based distortion patterns are largely subject-specific rather than driven by inherent features of the letters themselves (this outcome remained unchanged when A11 was excluded). Overall, the lack of consistent letter dominance—both within and across participants—suggests that perceptual distortions are mainly determined by *where* a letter appears in the VF rather than *which* letter is shown. In other words, the same spatial locations tend to produce distortions regardless of letter identity, supporting the idea that these distortions arise from localized, retinotopic irregularities in visual processing rather than from the visual features of specific letters.

### VA Difference and Letter Distortion

**Figure 6.**
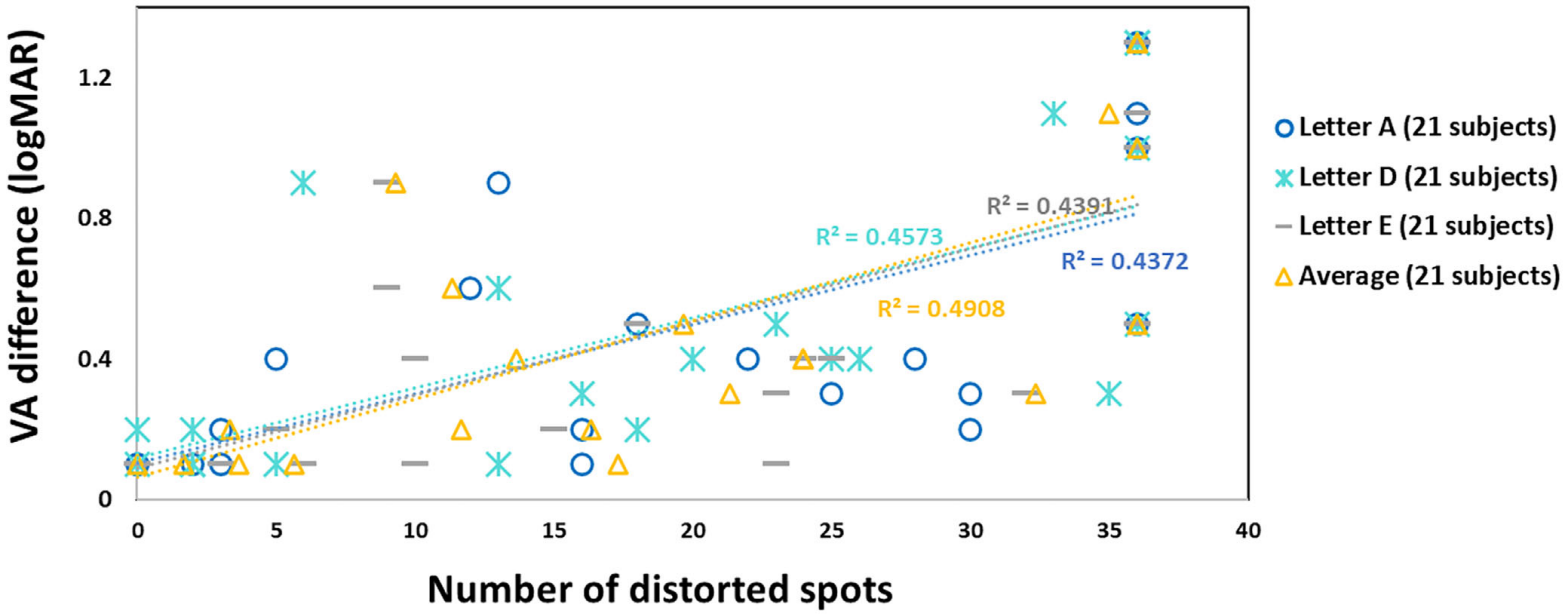
**Correlation between VA difference (logMAR) and letter distortion.** The figure illustrates the relationship between VA difference (*y*-axis) and the number of distorted spots (*x*-axis) for letters A, D, and E—distortion intensity—across subjects. Each letter is represented by 21 data points—one per subject. A significant positive correlation was observed on average (*R*^2^ = 0.4908, *P* < 0.001) and for each letter individually: A (*R*^2^ = 0.4573), D (*R*^2^ = 0.4391), and E (*R*^2^ = 0.4372).

The relationship between interocular VA difference (logMAR) and the average distortion intensity (average of all three letter types) across the subjects was strong and highly significant (*r* = 0.7, *P* < 0.001). This positive association was also observed for each letter individually—A (Spearman's rho = 0.72, *P* < 0.001), D (*r* = 0.67, *P* < 0.001), and E (*r* = 0.66, *p* < 0.001)—with similar correlation strength across letter types and the overall average (Fig. 6). These findings indicate that greater interocular acuity disparity is associated with more extensive letter distortion, suggesting that the severity of visual loss may influence the extent of perceived distortion when both are assessed using letter stimuli.

## Discussion

This study is the first, to our knowledge, to map letter distortions across the VF in individuals with amblyopia. Over 95% of participants exhibited stable and detectable letter distortions—a markedly higher prevalence than that observed for other distortion types in our previous study,^13^ which used simpler stimuli such as blobs and Gabors. This difference, found despite using the same participants, methods, and experimental setup as our previous work, suggests that letter distortion may offer a more sensitive and robust assay of perceptual distortion in amblyopia.

These findings highlight the critical role of stimulus selection in evaluating visual distortions. Different stimulus types—targeting SF, position, orientation, or letter form—can yield markedly different prevalence rates. Given its high prevalence and the direct relevance of letters to everyday visual tasks, letter distortion may serve as a valuable clinical tool for identifying and tracking visual dysfunction in amblyopia.

The letter distortion task was easy for participants to learn and quick to administer, requiring only brief familiarization and minimal instruction. Given the robustness and reliability of the measurements, the task could be adapted into a streamlined format suitable for clinical settings. In practice, clinicians could use letters of comparable size to those in standard acuity charts and present them individually at central fixation, asking patients to report any perceived shape irregularities. Such a simplified version could provide a rapid and accessible way to screen for spatial distortions in amblyopia and monitor changes over time.

These findings also resonate with earlier work by Hess and colleagues,^58^^–^^62^ who proposed that amblyopic distortions arise from spatial disorganization or irregular retinotopic sampling across the VF rather than from elevated internal noise or undersampling, as discussed by Levi and colleagues.^35^^,^^37^^,^^63^ The strong within-subject stability and spatial specificity observed here are consistent with this spatial-map framework, suggesting that local irregularities in retinotopic representation may underlie the reported distortions.

We also found that letter distortion patterns were highly reliable and consistent across the VF within individual subjects. Specifically, the spatial distribution of distortion for different letters (A, D, and E) showed strong within-subject similarity, suggesting that letter distortion is largely invariant to letter form. This consistency supports the idea that perceptual distortion in amblyopia may arise from localized VF deficits rather than from differences in stimulus-specific processing—especially considering that, in a previous study,^31^ regions of distortion in synthesized images closely overlapped with areas of position distortion mapped using blobs.

Interestingly, this finding contrasts with our previous study,^13^ in which different types of distortion—position, orientation, and SF—did not exhibit correlated spatial patterns within individuals. Each distortion type appeared to follow its own distinct topography, suggesting that separate underlying mechanisms may drive these perceptual anomalies. To address the apparent discrepancy between the findings of this and our previous study, three interpretations may be considered.

First, the consistent spatial distortion patterns across different letters suggest that letter-based distortions may belong to a single perceptual category. Letters share common visual properties such as defined shape and structural form and are likely processed along similar visual pathways.^64^^–^^68^ This could explain why distortions observed for different letters (e.g., A, D, E) are spatially aligned within individuals. In contrast, other distortion types—such as position, orientation, and SF—may each represent distinct categories, with unique underlying mechanisms and less interrelated spatial patterns. In our earlier study, the distortion maps for these categories were uncorrelated within the same subject, reinforcing the idea of multiple independent distortion mechanisms.

Second, it is possible that the nature of the distortion map depends more on the perceptual feature being probed than on the exact stimulus used. For example, assessing position distortion using blobs or small squares might yield similar spatial patterns, as both stimuli target positional accuracy. Likewise, probing orientation distortion with lines rather than Gabors could produce comparable results, as both stimuli emphasize orientation perception. In this study, although the stimuli were letters, the task was to detect form-related distortions, not specific features such as SF or orientation. This suggests that spatial distortion maps may remain stable across variations in stimulus design, as long as the *perceptual attribute* being tested remains consistent. However, changing the perceptual focus (e.g., from position to SF) may result in entirely different spatial distortion patterns. A similar view was expressed by Barrett and colleagues,^14^^,^^69^ who noted that different measurement paradigms—particularly those relying on subjective reports versus objective quantification—may engage distinct underlying mechanisms of distortion. This distinction may explain why related tasks can reveal different but complementary aspects of perceptual irregularity.

Third, the letter distortion task used a binary judgment: Participants reported whether a letter looked distorted or not, without describing the type or distortion intensity. This is different from our previous study,^13^ where distortions in position, SF, and orientation were tested using stimuli designed to target specific visual features. In that work, each task focused on one clear type of distortion—like shifting the location of a blob or changing the orientation or frequency of a Gabor. In contrast, distortions in letters can affect many different aspects at once, such as size, shape, alignment, or structure. These aspects often overlap in how people see them, which makes it difficult to separate or measure them individually. Because of this, the binary task in the current study had a different but purposeful goal: It allowed for fast, practical judgments that focused on whether distortion was present, rather than identifying what kind of distortion it was.

Participants in similar studies may notice that a letter appears “off” or wrong, but reporting whether it looks stretched, shrunken, displaced, or altered in some combination can often be difficult—not only for the participant to describe but also for the participant to demonstrate or reconstruct using visual elements when asked. For this reason, we used a binary approach that recorded whether distortion was detected at each location. The resulting letter distortion maps show where distortions were seen across the VF. Importantly, by having these spatial maps, we can begin to explore whether letter distortion follows a systematic pattern—one that may remain stable across different letters or within specific areas of the VF.

The reliability of these binary distortion maps was confirmed using the ICC, which quantifies the agreement of repeated measurements regardless of data type. In this context, a high ICC indicates that the same VF locations were consistently reported as distorted or undistorted across sessions, confirming the reproducibility of the maps. However, because the present method relies on binary judgments, it captures only whether distortion is detected—not how it appears or how strongly it is perceived. Subtle changes in the qualitative appearance of distortions may therefore go unnoticed. Although this design ensured simplicity and reliability, future studies could adopt graded response scales or reconstruction-based tasks to measure the appearance and intensity of distortions more precisely.

A limitation of this approach is that the distortion mapping relied on subjective reports of “any detected difference,” which could vary across participants. However, the purpose of this task was not to measure detection thresholds or distortion intensity, but rather to capture the *spatial organization* of perceived distortions across the VF. Although individual response criteria might influence the absolute number of reported distortions, the overall spatial structure of the maps was highly consistent across sessions and aligned with known spatial bias patterns in amblyopia. This consistency suggests that the observed distortions primarily reflect perceptual rather than decisional factors.

The types of distortions observed in this study share similarities with those reported previously. Barrett and colleagues,^14^^,^^69^ using Gabor patterns, described local changes such as missing or faded segments, wavy or fuzzy lines, and variations in line thickness. Although our stimuli were letters rather than gratings, participants in our study reported comparable effects, including partial disappearance of letter parts or uneven boldness of strokes. These similarities suggest that both tasks capture related forms of local contour distortion. Likewise, Pugh's work showed that distortions could appear consistently across different letter types, indicating that they do not depend on letter identity—a finding that aligns with our results.^18^^,^^19^ However, unlike Pugh's uniform chart-wide distortions, our data show spatially localized distortions that varied across the VF, highlighting that perceptual irregularities in amblyopia are position dependent rather than homogeneous. Together, these interpretations help reconcile the divergence in results across the two studies and emphasize the importance of both stimulus type and task goal in shaping perceptual distortion maps.

Despite the strong within-subject consistency of distortion patterns, these patterns were not consistent across subjects. Correlation analyses between the same letter maps across different individuals (e.g., letter A in subject 1 compared to letter A in subject 15, 20, or 10) revealed no meaningful spatial correspondence. This lack of between-subject correlation—observed independently for the letter A, D, and E maps—reinforces previous reports describing the highly idiosyncratic nature of perceptual distortions in amblyopia.^8^^,^^13^^,^^14^^,^^32^^,^^33^^,^^35^^,^^70^ However, as noted earlier, individual differences in response criteria may partly contribute to the low cross-participant correlations and should be considered when interpreting these results. Our findings demonstrate that the well-documented heterogeneity of amblyopic distortions also extends to letter-form perception—a domain that captures the functional demands of everyday vision. This subject-specific patterning emphasizes that perceptual distortions in amblyopia are best understood and evaluated through individualized, rather than generalized, assessment approaches.

Interestingly, we found no systematic dominance of one letter being more distorted than others, either within individual subjects or across the sample. This lack of letter-specific variability suggests that “letter distortion” reflects a unified perceptual deficit, rather than three separate distortions tied to the forms of A, D, or E. Therefore, the findings that (1) distortion maps are spatially aligned across letters within individuals, and (2) no single letter is disproportionately distorted—based on the number of distorted locations, which indicates the intensity of distortion regardless of its spatial pattern—both point toward a spatially organized, subject-specific pattern of distortion rather than a global form- or shape-based disruption.

That said, this interpretation warrants further investigation. Future studies should include a broader range of letters to determine whether the observed patterns are consistent across letters or whether certain groups of letters share distinct spatial distortion patterns. It remains possible that letters may fall into perceptual categories—each associated with a characteristic distortion map—based on shared visual features or structural complexity.

Another key finding was the positive correlation between letter distortion and VA loss. Although our previous study^13^ using blobs and Gabors found no such relationship, the present results show a significant association between the distortion intensity and the magnitude of VA difference. This divergence suggests that different distortion types may be driven by distinct neural mechanisms and may vary in their clinical relevance; here, this relationship was evaluated cross-sectionally based on current distortion and acuity measures.

Importantly, although letter distortion correlates with VA, the two measures capture different aspects of visual function. VA assesses recognition accuracy—whether a letter can be correctly identified—whereas our letter distortion detection task reveals qualitative anomalies in how that letter appears. A letter might be distorted (e.g., stretched, compressed, or shifted) yet still be accurately recognized, highlighting a dissociation between perceptual clarity and identification ability. This distinction emphasizes the complementary nature of these assessments—although evaluating VA remains essential, letter distortion offers unique insights into the perceptual experience of individuals with amblyopia.

### Future Work

The present design focused on mapping the presence or absence of distortion rather than quantifying detection thresholds. Future studies could incorporate threshold-based or forced-choice paradigms to better characterize response bias and measure distortion sensitivity across VF locations. The hand-drawn reconstructions of perceived distortions collected in this study also represent a valuable qualitative dataset. Future work could digitize these drawings to quantify geometric similarities among distorted letter shapes—for example, by assessing their overlap with canonical letter templates or by using image-based metrics such as Dice coefficients—to explore common structural features across locations or participants.

Earlier work by Sireteanu et al.^31^ also emphasized the complexity of linking subjective perceptual experience to objective reconstructions of distortion. Their observations of mismatches between what amblyopic observers perceived and what could be modeled computationally highlight the challenges of translating perceptual anomalies into objective visual representations.

Building on these findings, future studies could further characterize the spatial distribution of distortions across the VF. Although the present study was not designed to quantify distortions as a function of eccentricity, examining whether distortions occur more frequently near the center or the periphery could provide additional insight into how spatial location influences perceptual irregularities. Such work would help clarify whether distortion patterns align with known variations in visual sensitivity and cortical magnification across eccentricities.

Although readability was not directly examined in this study, it represents an important direction for future research. Here, we intentionally used easily recognizable letters (A, D, and E) to minimize differences in legibility and to focus solely on mapping perceptual distortions. Nonetheless, readability and distortion share overlapping perceptual components, and letter form complexity could influence how distortions are perceived and how they impact reading performance. Future studies could systematically vary letter complexity or familiarity to compare distortions across easy- and difficult-to-read letters, thereby extending the current approach to a broader domain linking perceptual distortion and functional readability.

## Conclusions

Our findings identify letter distortion as a sensitive, spatially organized, and clinically meaningful indicator of visual dysfunction in amblyopia. Its high prevalence and strong correlation with VA loss underscore its potential for diagnostic use, individualized assessment, and tracking treatment outcomes. Future studies should build on these findings by testing a broader range of letters and further examining the neural and perceptual mechanisms underlying letter-based distortions.
